# Two decades of association mapping: Insights on disease resistance in major crops

**DOI:** 10.3389/fpls.2022.1064059

**Published:** 2022-12-06

**Authors:** Sunil S. Gangurde, Alencar Xavier, Yogesh Dashrath Naik, Uday Chand Jha, Sagar Krushnaji Rangari, Raj Kumar, M. S. Sai Reddy, Sonal Channale, Dinakaran Elango, Reyazul Rouf Mir, Rebecca Zwart, C. Laxuman, Hari Kishan Sudini, Manish K. Pandey, Somashekhar Punnuri, Venugopal Mendu, Umesh K. Reddy, Baozhu Guo, N. V. P. R. Gangarao, Vinay K. Sharma, Xingjun Wang, Chuanzhi Zhao, Mahendar Thudi

**Affiliations:** ^1^ Crop Genetics and Breeding Research, United States Department of Agriculture (USDA) - Agriculture Research Service (ARS), Tifton, GA, United States; ^2^ Department of Plant Pathology, University of Georgia, Tifton, GA, United States; ^3^ Department of Agronomy, Purdue University, West Lafayette, IN, United States; ^4^ Dr. Rajendra Prasad Central Agricultural University (RPCAU), Bihar, India; ^5^ Indian Council of Agricultural Research (ICAR), Indian Institute of Pulses Research (IIPR), Kanpur, Uttar Pradesh, India; ^6^ Crop Health Center, University of Southern Queensland (USQ), Toowoomba, QLD, Australia; ^7^ Department of Agronomy, Iowa State University, Ames, IA, United States; ^8^ Faculty of Agriculture, Sher-e-Kashmir University of Agricultural Sciences and Technology (SKUAST), Sopore, India; ^9^ Zonal Agricultural Research Station (ZARS), Kalaburagi, University of Agricultural Sciences, Raichur, Karnataka, India; ^10^ International Crops Research Institute for the Semi-Arid Tropics (ICRISAT), Hyderabad, Telangana, India; ^11^ College of Agriculture, Family Sciences and Technology, Dr. Fort Valley State University, Fort Valley, GA, United States; ^12^ Department of Plant Science and Plant Pathology, Montana State University, Bozeman, MT, United States; ^13^ Department of Biology, West Virginia State University, West Virginia, WV, United States; ^14^ International Maize and Wheat Improvement Center (CIMMYT), Nairobi, Kenya; ^15^ Institute of Crop Germplasm Resources, Shandong Academy of Agricultural Sciences (SAAS), Jinan, China

**Keywords:** plant diseases, genome wide association studies, haplotypes, pangenomes, multi-parent populations, *k*-mers

## Abstract

Climate change across the globe has an impact on the occurrence, prevalence, and severity of plant diseases. About 30% of yield losses in major crops are due to plant diseases; emerging diseases are likely to worsen the sustainable production in the coming years. Plant diseases have led to increased hunger and mass migration of human populations in the past, thus a serious threat to global food security. Equipping the modern varieties/hybrids with enhanced genetic resistance is the most economic, sustainable and environmentally friendly solution. Plant geneticists have done tremendous work in identifying stable resistance in primary genepools and many times other than primary genepools to breed resistant varieties in different major crops. Over the last two decades, the availability of crop and pathogen genomes due to advances in next generation sequencing technologies improved our understanding of trait genetics using different approaches. Genome-wide association studies have been effectively used to identify candidate genes and map loci associated with different diseases in crop plants. In this review, we highlight successful examples for the discovery of resistance genes to many important diseases. In addition, major developments in association studies, statistical models and bioinformatic tools that improve the power, resolution and the efficiency of identifying marker-trait associations. Overall this review provides comprehensive insights into the two decades of advances in GWAS studies and discusses the challenges and opportunities this research area provides for breeding resistant varieties.

## Introduction

The incidence and severity of biotic and abiotic stresses have been increasing due to global climate change. Plant diseases have been serious threat to global food security as well as devastating in the history of mankind which led to famines and mass migration of humans. For instance, ancient Israelites migrated to Egypt due to incidence of wheat rust. Similarly, the ergot of rye, destroyed the armies of Peter the Great at Astrakhan in 1722, while the late blight of potatoes led to Irish famine (Goss et al., 2014). Potato blight laid waste the economy of Ireland in the 1840s, and led to migrations that changed the history of the New World. In 1943 brown spot disease (caused by *Helminthosporium oryzae*) resulted in the Bengal famine that took lives of millions of people (Islam, 2007). Frequent droughts, higher temperatures and other abiotic stresses cause biochemical and physiological changes in plants that increase their vulnerability to diseases and also led to emergence of new races or pathotypes. Coffee rust destroyed the coffee-trees of Ceylon in the 1880s and caused the economy of the England to be switched to tea-growing. Global yield losses in major crops due to plant disease is around 30% (Rizzo et al., 2021) and average yield losses in five globally important crops by plant pathogen estimates at a global level; rice (30.0%), wheat (21.5%), maize (22.5%), soybean (21.4%) and potato (17.2%) suggest that the highest losses are associated with resource-poor regions with growing populations (Savary et al., 2019). In other words, the resource-poor farmers have been most adversely affected by the yield loss which increased their debts making them more vulnerable to food, health and educational security.

Although conventional disease management strategies like chemical control have reduced the yield losses caused by plant pathogen, but the pesticide formulations lead to extreme deterioration of the soil and environment. Further, traditional breeding for disease resistance is time consuming and labor intensive (Deng et al., 2020). Over the last two decades, advances in genomics and next-generation sequencing technologies enabled the development of enormous genomic resources like genomes of crops (Michael and Jackson, 2013; Kress et al., 2022) as well as pathogens (Moller and Stukenbrock, 2017). Several efforts at the international level have been made to gain insight into the disease resistance mechanism in case of several pathogens in different major crop plants. Quantitative trait locus (QTL) mapping has been widely used for identifying the genomic regions/genes associated to disease resistance traits. Further, crop varieties with enhanced resistance to key diseases have been developed (Dodia et al., 2019; Gangurde et al., 2019; Mannur et al., 2019; Pandey et al., 2020; Roorkiwal et al., 2020; Gangurde et al., 2021). Nevertheless, QTLs detected using the linkage mapping approach are sometimes not deployable as large genomic regions associated with linkage drag or undesirable genes. Association mapping that overcomes the limitations of QTL mapping has been used in several crop plants (see Alseekh et al., 2021) and animal species (Sharma et al., 2015; Uffelmann et al., 2021) for fine mapping in identifying markers associated with the traits of interest. Although GWAS was started in animals, humans, and the perennial tree species, wherein it isn’t easy to have biparental kind of genetic populations, nevertheless, it gained momentum in most crop species. The last decade has also witnessed huge progress in testing new GWAS models to achieve better and more precise results, including the implementation significance level to check the false discovery rate (Weckwerth et al., 2020).

In this article, we comprehensively review the progress of candidate gene discovery for disease resistance in major crop plants. In addition, we also provide insights into the use of multi-parental mapping populations for establishing genome-wide association studies and the extreme phenotype genome-wide association study (XP-GWAS), which does not require genotyping of a large number of individuals, and which further reduces the cost, labor and time involved. The statistical basis of genome screening is the most important part of GWAS, as statistical tools are used to predict the correct association of outcomes by calculating a large amount of data. Power and resolution are the two important factors that can alter the genome-wide association. Power represents the ability to detect an association, and resolution regards the proximity of the association between a marker and quantitative trait locus (Mohammadi et al., 2020). In multiple-marker association, we have presented an alternative to the statistics of single-marker association. The combination of methods is the most desired approach, as multiple combinations of methods will discover more signals across the genome. Tools and software are the most important pillars for genome-wide association analysis, and some important tools and software will be discussed in this review. Additionally, we will focus on advances in GWAS analysis and the future outlook for association mapping.

## Marker-trait associations (MTAs) and candidate gene discovery disease resistance in crops

Association mapping is widely used to check marker associations with a specific trait based on the difference in allele frequency across the genomes (Uffelmann et al., 2021). More precisely, it is a powerful tool for the genome-wide detection of genomic regions/candidate genes for complex traits over the time-consuming and imprecise QTL mapping approach. Detected genomic regions will provide information on unrelated individuals to elucidate the molecular basis of biotic and abiotic stress tolerance. Candidate genes identified by non-random association of alleles in GWAS can be used to accelerate breeding programs to develop new varieties. Researchers have done considerable work to make biotic and abiotic stress-tolerant varieties. GWAS requiring high-density genome-wide markers and SNPs based on next-generation sequencing have been widely used for dissecting complex agronomic traits and disease resistance loci in economically important crop plants (Ogura and Busch, 2015; Burghardt et al., 2017). In this review, we mainly focus on and demonstrate studies that have used GWAS approaches in various crops to understand disease resistance responses.

## Maize

Maize (*Zea mays*) is one of the widely cultivated major crops across the globe. Despite wide investigations and availability of genome sequences, very few studies deployed candidate gene-based GWAS to identify candidate genes for disease resistance in maize. In the seminal study, 18 novel genes associated with head smut resistance were identified (Wang et al., 2012). *ZmFBL41* gene encoding F-box protein that confirms resistance to sheath blight and banded leaves was reported using GWAS (Li et al., 2019a). Dwarf disease resistance in maize was investigated by integrating GWAS and linkage mapping, and candidate genes identified by GWAS include DRE-binding protein (*GRMZM2G006745*) and LRR receptor-like serine/threonine-protein kinase (*GRMZM2G141288*) (Zhao et al., 2022a). A total of 10 MTAs for gray leaf spot resistance explaining ~15.7% phenotypic variance were identified (Kibe et al., 2020). In total, 164 significant associations with 25 candidate genes identified for *Fusarium verticillioides* resistance (Stagnati et al., 2019). A total of 17 significantly associated haplotypes in genomic regions of important candidate genes *Ht2*, *Ht3* and *Htn1* were identified for Northern corn leaf blight resistance (Rashid et al., 2020). Fumonisin is a mycotoxin produced in maize kernals, 17 MTAs and important candidate genes associated with fumonisin resistance were identified (Samayoa et al., 2019) (**Table 1**).

**Table 1:** Summary of MTAs or candidate genes identified for disease resistance in major crops.

Crop	Disease	Panel size	Candidate gene/QTL/MTAs	PVE (%)	Reference
Maize	Head smut	144	18 MTAs	3-9	Wang et al. (2012)
	Northern corn leaf blight	999	22 (12 + 10) MTAs	2-3	Ding et al. (2015)
	Leaf and sheath blight	318	28 (*ZmFBL41*)		Li et al. (2019a)
	Rough dwarf	292	22 MTAs		Zhao et al. (2022a)
	Gray leaf spot (GLS)	410	10 MTAs	15.7	Kibe et al., 2020
	*Fusarium verticillioides*	265	164 MTAs, 25 candidate genes		Stagnati et al., 2019
	Northern corn leaf blight	419	17 haplotypes, *Ht2*, *Ht3* and *Htn1* genes		Rashid et al., 2020
	Fumonisin resistance	256	17 MTAs		Samayoa et al., 2019
Rice	Bacterial leaf blight	285	*qPXO79_6-1 to -4*	11-29	Dilla-Ermita et al. (2017)
			qPXO339/349_9-1 and		
			qPXO339/349_11-1,		
			qPXO79/112/341_12-1		
		259	58 candidate genes,		Shu et al. (2021)
			*LOC_Os07g02560* and *LOC_Os07g02570*		
		895	77 loci	4-30	Jiang et al. (2021)
		421	13 QTLs		
	Bacterial leaf streak	895	7 loci		Lu et al. (2020)
		236	12 QTLs	–	Sattayachiti et al. (2020)
	Blast	151	21 MTAs	–	Wang et al. (2015)
		234	56 QTLs, *Pikx*		Li et al. (2019b)
		311	14 MTAs	74	Volante et al. (2020)
		331	3 QTLs, NIS1, RRobN1 NIS2 and NIS3	–	Frontini et al. (2021)
		355	127 MTAs	30	Lu et al. (2019)
					
	Sheath blight	217	10 MTAs	–	Jia et al. (2012)
		563	134, 562, and 75 MTAs	–	Zhang et al. (2019a)
		299	*qSB-3* and *qSB-6*	–	Chen et al. (2019)
		240	qLN1128 qMLL1114	18	Oreiro et al. (2020)
		259	1396 loci, 653 genes	–	Wang et al. (2021)
Wheat	Yellow rust	419	7 QTLs	6-19.9	Ledesma-Ramírez et al. (2019)
	Stripe rust	94	31 MTAs	11.9	Muhammad et al. (2020)
		240	12 MTAs	3.6 - 10.3	Jia et al. (2020)
	Leaf rust, tan spot and yellow rust	333, 313	36 (12 + 14+10) MTAs	–	Juliana et al. (2018)
	Fusarium head blight	273	10 MTAs	–	Arruda et al. (2016)
		170	9 MTAs	7.2-13.2	Wang et al. (2017)
		171	88 MTAs	6.6-14.8	Hu et al. (2020)
		240	5 MTAs	5.4-10.3	Zhu et al. (2020)
Barley	Spot blotch	261	23+15 QTLs	26-76	Visioni et al. (2020)
	Barley yellow dwarf virus	335	36 MTAs		Choudhury et al. (2019)
Sorghum	Downy mildew	368	16 genetic variants	26.7	Rashid et al. (2018)
	Target leaf spot	456	*flg22* and Chitin		Samira et al. (2020)
	Anthracnose	335	3 MTAs	–	Cuevas et al. (2018)
		359	16 MTAs	–	Prom et al. (2019)
		242, 163 and 159	24 (8 + 0 + 16) MTAs		Ahn et al., 2019; Ahn et al., 2021 and Ahn et al., 2022
	Grain mold	635	*KAFIRIN* and *LEA3*		Nida et al. (2019)
Soyabean	Tobacco ringspot virus	19652	2 chromosome		Chang et al. (2016)
	Stem rot disease	478	44 MTAs		Rolling et al. (2020)
	Sclerotinia stem rot	185	16 QTN		Sun et al. (2020)
	Soybean mosaic virus	219	24 MTAs	25.5–33.6	Che et al. (2020)
Peanut	Early leaf spot, late leaf spot and tomato spotted wilt	120	87 (18 + 28 + 41) MTAs	10.1-24.1	Zhang et al. (2019b)
	Root-knot nematodes	161	46 MTAs	7.8- 17	Kumral (2019)
	*Aspergillus flavus*	99	60 MTAs	16.8- 31.7	Yu et al. (2020)
*Brassica napus*	*Leptosphaeria maculans*	179	694 MTAs (*Rlm12*)		Raman et al. (2016)
		421	59 MTAs	2.9-23.4	Raman et al. (2020)
		585	79 MTAs	–	Fikere et al. (2020)
		243	25 resistance gene analogs	–	Fu et al. (2020)
	Clubroot	427	9 MTAs	4.2-6.5	Li et al. (2016a)
	Sclerotinia stem rot	448	26 MTAs to *DSRC4*, DSRC6 and DSRC8 loci	6.14	Wu et al. (2016)
				
Chickpea	Ascochyta blight	69	100 kb region (AB4.1) on chromosome 4	*-*	Li et al. (2017a)
		251	26 MTAs	11.4-25.9	Raman et al. (2022)
		165	30 MTAs		Farahani et al., 2022
	*Pythium ultimum*	184	11 MTAs, 7 candidate genes		Agarwal et al., 2022
					
Cotton	Verticillium wilt and Fusarium wilt	376	28 (15 + 13) MTAs	11-45	Abdelraheem et al. (2020)
	Verticillium wilt	299	17 MTAs	–	Li et al. (2017b)
Potato	*Phytophthora infestans*	150	16 QTLs	13.7-50.9	Juyo et al. (2019)
					
Tomato	Bacterial wilt	191	8 MTAs	8.3-18.2	Nguyen et al. (2021)
					
Common bean	Fusarium wilt	205	11 significant SNPs	9-64	Paulino et al. (2021)
Cassava	Cassava mosaic	6128	One major QTL	–	Wolfe et al. (2016)
	Cassava mosaic	5130 clones	3 MTAs	–	Rabbi et al. (2020)
Peach	Gummosis	195	5 MTAs	–	Li et al. (2021b)

PVE, Percent phenotypic variation explained; MTA, marker-trait associations.

## Rice

Rice (*Oryza sativa*) is staple food for more than half of the global population, and second most important cereal after Maize. In the case of rice, fungal diseases like blast, sheath blight and sheath-rot, bacterial diseases like bacterial blight (BB) and the viral disease like rice tungro disease, are major diseases. GWAS approach has been deployed to identify and validate genomic regions for tolerance to BB (Jiang et al., 2021; Shu et al., 2021), bacterial leaf streak tolerance (Sattayachiti et al., 2020; Jiang et al., 2021), blast (Volante et al., 2020; Frontini et al., 2021). Among 56 important QTLs/genomic regions associated with different blast isolates, a single genomic region was designated as the *Pik* allele that confirms resistance to all three isolates (Li et al., 2019b). Fourteen marker trait associations (MTAs) for blast resistance were identified using both field and growth chamber screenings by evaluating 311 *O. sativa* accessions (Volante et al., 2020), however, three novel regions (*BRF10*, *BRF11–2* and *BRGC11–3*) were identified that had no relationship with previously identified genes or QTLs. In rice, high nitrogen input levels are conducive to disease development, a phenomenon called nitrogen-induced susceptibility. Two important QTLs include *NIS2* and *RRobN1* were identified that may play an important role in the blast disease response to nitrogen fertilizer (Frontini et al., 2021). There are only a few reports about the identification of sheath blight (ShB) resistance QTLs using GWAS (Jia et al., 2012; Zhang et al., 2019a; Oreiro et al., 2020; Wang et al., 2021) (**Table 1**). A genomic region (*qLN11* and *qMLL11*) controlling sheath blight resistance was investigated recently (Oreiro et al., 2020). Additionally, GWAS with 259 diverse rice varieties identified 653 significantly associated with ShB resistance and validated two important disease resistance proteins, RPM1 (*OsRSR1*) and protein kinase domain-containing protein (*OsRLCK5*) (Wang et al., 2021). Transgenic rice containing the overexpressed *NH1* gene acquired high levels of resistance to *Xanthomonas oryzae* (Chern et al., 2005). This shows the importance of identifying and interrogating high-yielding varieties to better resist the disease. Rice panicle blast resistance gene, *Pb2*, encoding NLR Protein was reported recently (Yu et al., 2022)

## Wheat

In the case of wheat (*Triticum aestivum*), genetic loci for disease resistance to yellow rust (Ledesma-Ramírez et al., 2019), stripe rust (Juliana et al., 2018; Jia et al., 2020; Muhammad et al., 2020) and Fusarium head blight have been reported using GWAS approach (Arruda et al., 2016; Wang et al., 2017; Hu et al., 2020; Zhu et al., 2020). Further, genetic loci associated with resistance to multiple diseases such as leaf rust, stripe rust, and tan spot were also identified (Juliana et al., 2018). Using pre-breeding lines Ledesma-Ramírez et al. (2019), reported 14 SNP loci associated with seven genomic regions for yellow rust resistance. Similarly, among 12 stable loci reported to be associated with yellow rust resistance, six loci were novel and six were same as reported earlier using QTL studies (Jia et al., 2020) (**Table 1**). Using 171 wheat cultivars, two syntenic loci, *QFhb-4AL* and *QFhb-5DL*, associated with Fusarium head blight resistance were reported (Hu et al., 2020). Using genotyping-by-sequencing SNPs, 10 MTAs Fusarium head blight resistance and few SNPs associated with *Fhb1* on chromosome 3B were reported (Arruda et al., 2016). High-resolution SNP-based GWAS enabled identification of 19 stable genomic regions harboring 292 significant SNPs associated with adult-plant resistance and rapid identification of putative resistance genes and can be used to improve the efficiency of marker-assisted selection in wheat disease resistance breeding (Wu et al., 2021). Recently, stable and environment-specific QTLs for powdery mildew (PM) adult-plant resistance were identified on chromosomes 1A, 1B, 1D, 2B, 3B, 4A, 5A, 6A, and 6B for *Septoria tritici* blotch and 2A, 2D, 3A, 4B, 5A, 6B, 7A, and 7B (Alemu et al., 2021). Four novel QTLs strongly associated with different markers for barley yellow dwarfism in wheat were identified on different chromosomes (Choudhury et al., 2019).

## Barley

In case of barley (*Hordium vulgare*), rusts and PM diseases that have a major effect on yield. Based on phenotyping of 431 European barley accessions for two seasons and genotyping using DArT-seq, 78 MTAs for PM and rusts adult plant resistance were reported (Czembor et al., 2022). In case of spot blotch resistance, 11 out of 20 genetic loci at the seedling and adult stages were associated with functional candidate genes. Most of the identified genomic regions seem to be enriched with some known important proteins associated with disease resistance, such as NBS-LRR, transcription factors and pathogenesis-related proteins (Visioni et al., 2020). Using multi-location phenotyping of 1,317 spring barley breeding lines from a commercial breeding program and genotyping using 9K SNP array, a QTL on chr. 4H associated with PM and ramularia resistance were reported (Tsai et al., 2020).

## Sorghum

In case of sorghum (*Sorghum bicolor*) using association analysis genetic loci linked to various disease resistances including anthracnose, head smut, downy mildew, and target leaf spot, were reported (Cuevas et al., 2018; Samira et al., 2020; Ahn et al., 2021; Ahn et al., 2022 and Chaturvedi et al., 2022). Two SNPs on chromosome 9 that are linked to the *Sb09g029260* gene, a member of the chalcone and stilbene synthase family were reported (Adeyanju et al., 2015). Genomic regions containing important genes like YELLOW SEED1 (Y1), a non-functioning pseudogene (Y2), and YELLOW SEED3 (Y3) were found to be associated with grain mold resistance (Nida et al., 2019). The defense mechanism against leaf spot disease in sorghum was clarified by GWAS analysis, which also identified two SNP loci linked to flg22 and the chitin response (Samira et al., 2020). In majority of the cases, it has been found that leucine-rich repeat (LRR) region resistance genes are responsible for signal transduction in plants towards activating defense genes and form major class of R genes. LRR proteins have enormous functions including signal transduction, protein-protein interactions, and cell adhesion. Some of these mechanisms are overlapping between responses due to insect and pathogen induced. For example, several LRR proteins were highlighted including other compounds involved in defense responses (Punnuri et al., 2022). This functional adaptability of LRR proteins derives from a conserved three-dimensional structure, a curved coil composed of repeating units of ~24 amino acid residues, that contains both conserved and variable regions.

## Soybean

Yield in soybean (*Glycine max*) is adversely affected by a wide range of pathogens like fungi, bacteria, viruses, and nematodes. MTAs identified for various diseases are comprehensively reviewed recently by Ferreira and Marcelino-Guimarães (2022). GWAS identified a single locus on chromosome 2 strongly associated with tobacco ringspot virus sensitivity (Chang et al., 2016). A mapping study for stem rot disease resistance using genome-wide association study analysis was conducted and identified 44 QTLs for quantitative disease resistance (Rolling et al., 2020). A specific locus amplified fragment sequencing (SLAF-seq) approach was used to genotype for GWAS and identified seven genomic regions with major effects and nine novel regions with minor effects on Sclerotinia stem rot resistance (Sun et al., 2020). Many associated SNPs were identified that are tightly linked with previously reported SMV resistance loci, *Rsv1*, *Rsv4*, and *Rsv5* (Che et al., 2020).

## Peanut

Peanut (*Arachis hypogaea*) is an important oilseed crop with a large and complex genome, is one of the most nutritious food. A comprehensive GWAS study based on 300 genotypes peanut from 48 countries identified 1 MTA for *Aspergillus flavus* resistance, 6 MTAs for early leaf spot, 31 MTAs for groundnut rosette disease and 1 MTA identified for late leaf spot of peanut (Pandey et al., 2014). Early leaf spot (ELS) and late leaf spot (LLS) tomato spotted wilt virus (TSWV) are serious peanut diseases. In case of peanut, of 74 non-redundant genes identified as resistance genes, 12 candidate genes were in significant genomic regions including two candidate genes for both ELS and LLS, and other 10 candidate genes for ELS (Zhang et al., 2020). Similarly, a total of 22 non-redundant candidate genes were identified significantly associated with diseases, which include 18 candidate genes for TSWV, 3 candidate genes for both ELS and LLS, and 1 candidate gene for LLS, respectively (Zhang et al., 2019b). Most candidate genes in the associated regions are known to be involved in immunity and defense response. The QTLs and candidate genes obtained from this study will be useful to breed peanut for resistances to the diseases. Root-knot nematodes are also major problem in case of peanuts, 46 genetic loci with phenotypic variation explained (PVE) between 7.8% and 17% located on 12 different chromosomes underlying root-knot nematode resistance were determined by GWAS (Kumral, 2019). In GWAS of groundnut, a total of 60 significantly associated SNPs were identified with 16.87% to 31.70% phenotypic variation for resistance to *Aspergillus flavus* (Yu et al., 2020).

## Brassica sps

In Canola (*Brassica napus)* using GWAS analysis*, Rlm12* locus was reported to be associated with adult plant resistance to blackleg disease caused by *Leptosphaeria maculans* (Raman et al., 2016). Using Canadian and Chinese canola accessions, 32 and 13 SNPs loci distributed on chromosomes A03, A05, A08, A09, C01, C04, C05, and C07 that were tightly associated with blackleg resistance were reported (Fu et al., 2020). Recently, 133 SNPs associated with 123 loci for disease traits of sclerotinia stem rot were reported using GAPIT R package and GEMMA-MLM (Roy et al., 2021). Nine genomic regions were identified that showed a significant association with clubroot resistance by using GWAS of 472 accessions with Brassica 60K Infinium® SNP array (Li et al., 2016a). Similarly, three QTLs, *DSRC4*, *DSRC6*, and *DSRC8*, associated with Sclerotinia stem rot resistance were also reported (Wu et al., 2016).

## Chickpea

Chickpea (*Cicer arietinum* L.) is second most important grain legume cultivated in more than 150 countries across the globe. Fusarium wilt, Ascochyta blight (AB), and Botrytis grey mould are major diseases that lead to yield losses in chickpea growing regions. Association mapping approach was extensively deployed in case of abiotic stress (Thudi et al., 2014; Varshney et al., 2019). Very few studies reported the genetic loci and candidate genes associated with resistance to AB resistance. For instance, 26 genomic regions on chromosomes Ca1, Ca4, and Ca6 associated with AB resistance can be used in chickpea breeding programs to enhance AB resistance using marker-assisted/genomic selection strategies (Raman et al., 2022). In addition, a 100 kb region (*AB4.1*) on chromosome 4 with 12 predicted genes (like NBS-LRR receptor-like kinase, wall-associated kinase, zinc finger protein, and serine/threonine protein kinases) significantly associated with AB resistance was reported (Li et al., 2017a). Recently, association mapping discovered 11 significant MTAs and seven candidate genes for pre-emergence damping-off resistance in chickpea (Agarwal et al., 2022).

## Other key crops

In cotton (*Gossypium hirsutum*), genomic regions, NBS-LRR and enriched with resistance gene analog (RGA) clusters (*RGA1* and *RGA3*) associated with two different strains causing wilt disease (Abdelraheem et al., 2020). While, 17 significant SNPs and 22 candidate genes associated with verticillium wilt resistance were predicted by haplotype block structure analysis (Li et al., 2017a). Similarly, for BB 11 genomic regions associated with 79 SNPs found on different chromosomes were reported (Elassbli et al., 2021). In case of potato (*Solanum tuberosum*), 16 QTLs associated with resistance to late blight were reported, with PVE between 13.7% and 50.9%. Of 15 candidate genes found in the study, ten for stem resistance and five for leaf resistance were reported (Juyo et al., 2019). In the case of tomato (*Lycopersicum esculentum*), eight genomic regions associated with bacterial wilt resistance and their corresponding QTLs (*Bwr-4* and *Bwr-12*) explaining 8.36–18.28% PVE were identified (Nguyen et al., 2021). In case of common bean, the molecular basis of fusarium wilt resistance was elucidated; significant SNPs and candidate genes related to carboxy-terminal LRR and nucleotide-binding sites were reported (Paulino et al., 2021). In cassava, fourteen genomic regions were identified, among which a single region on chromosome no. 8 account for 30 to 66% of genetic resistance to mosaic disease resistance (Wolfe et al., 2016). A total of 29 MTAs on chromosome 10 and SIN_1019016 one of the candidate genes identified closely associated with phytophthora blight resistance in sesame (Asekova et al., 2021). Two genomic regions on chromosomes 2 and 9 of *Setaria italica* were significantly associated with blast disease resistance in foxtail millet (Li et al., 2021a). In vegetable and fruit crops the GWAS was very extensively used for candidate gene discovery and development of diagnostic markers. Diverse set of 566 apple accessions identified significant marker trait associations for fire blight of apple caused by *Erwinia amylovora*. A total of 23 and 38 MTAs significantly (p<.001) associated with shoot and blossom blight resistance, respectively (Thapa et al., 2021). GWAS based on 195 accessions and 145,456 genome-wide SNPs identified five SNPs and six candidate genes significantly associated with gummosis disease resistance in peach (Li et al., 2021b). In *Brassica napus* genome-wide association analysis based on association panel of 448 accessions genotyped with the Brassica 60K Infinium^®^ SNP array identified 26 SNPs corresponding to three loci, DSRC4, DSRC6, and DSRC8 were associated with Sclerotinia stem rot resistance (Wu et al., 2016).

## Statistical basis of genome screening

In mathematical terms, GWAS analysis consists a series of statistical testing, screening the genome with one marker at a time, one region at a time, or the whole genome at once. The hypotheses under evaluation consist of the null hypothesis (*H*_0_) and the alternative hypothesis (*H*_1_). Under *H*_0_, the marker under evaluation is not associated with the trait, whereas the alternative hypothesis rejects *H*_0_. The consensus metric of association is the p value, defined as the probability of observing the association informed by the data given that the null hypothesis is true. Thus, lower values support the rejection of *H*_0_. The preferred scale for p value is -log_10_(p value), so that stronger associations are displayed as higher values. The genome-wide plot where the markers are ordered according to their physical position on the x-axis with associations presented on the y-axis in terms of -log_10_ (p value) is referred to as the Manhattan plot.

When a single marker is tested, the target p value to assert an association is equal to or lower than *α* = 0.05, which allows spurious associations 5% of the time. However, genome-wide screening entails testing thousands to millions of markers, and consequently, there is an expectation of 5% false discoveries. To mitigate false positives, the significance threshold is adjusted to account for the multiple testing problem (*m*). A standard procedure is the Bonferroni correction, which consists of dividing *α* by the number of markers, creating a more stringent threshold to define an association. The Bonferroni threshold may be too stringent with sequence-level data involving millions of markers. A threshold relaxation is attained through an acceptable false discovery rate (*FDR*), referred to as Benjamini–Hochberg, which consists of dividing *α* by (1-*FDR*) × *m*. Other alternatives include replacing the total number of markers by the effective number of segments (*m* ^*^), which accounts for marker collinearity associated with linkage disequilibrium.

## Approach 1: Single-marker associations

Linear models represent the main framework to test marker-QTL associations on complex traits. At its simplest form, the linear model that defines the alternative hypothesis, ergo fitting a marker, is


y=μ+xb+e

where **y** is the vector of phenotypes, **μ** is the intercept, **x** is the vector containing the marker information, b is the marker effect, and **e** is the vector of residuals. The marker information is normally coded as {0,1,2} corresponding to {AA,Aa, aa} to capture the additive effect of an allele substitution. The null hypothesis model does not contain a marker term and is defined as


y=μ+e

In both cases, the residuals are assumed to be normally and identically distributed as $e∼\text{N(}0,I\sigma_{\text{e}}^{2})$ . The likelihood and null and alternative models are defined by


$$\text{L}(\text{X|}b,V)=\frac{\exp{\left(y-Xb\right)}^{\prime }V^{-1}(y-Xb)}{\sqrt{2\text{π}\left|V\right|}}$$

where **b** is a vector of fixed effect coefficients, including the intercept and the marker, and **V** is the variance-covariance matrix, which for this simple model $V=I\sigma_{\text{e}}^{2}$ . Adequate statistical testing is the likelihood ratio test (LRT) between null and alternative models. It contrasts the likelihood of the data with and without the marker in the model, hence measuring the improvement in data fit when the marker under evaluation is included in the model. The test is defined by


$$\text{LRT}=-2\ln\left(\frac{L_{H0}}{L_{H1}}\right)$$

The p value is obtained from LRT from chi-squared density with the number of degrees of freedom (*v*) dictated by the difference in degrees of freedom from models *H*_0_ and *H*_1_; thus, LRT ~x^2^(*v*). For the simple case presented above that corresponds to one degree of freedom because there is one additional parameter in the alternative model, that is, the marker effect (b). When the single marker association jointly tests for additive and dominant effects, the LRT is tested with two degrees of freedom, which may reduce the statistical power.

## Power and resolution

Two important factors influencing the outcome of the genome-wide association are the power and resolution of the analysis. Power corresponds to the ability to detect an association and resolution regarding the proximity of the association between the marker and quantitative trait locus (Mohammadi et al., 2020).

The influence of power on signal detection is known as the Beavis effect (Beavis, 1998; Xu, 2003). Power can be increased with (1) an increasing number of phenotypic observations; (2) the imputation of missing marker information (Xavier et al., 2016a), as it increases the number of marker observations; (3) a good experimental design that increases the genetic signal; and (4) the design of a recombinant population with higher minor allele frequency and SNP variance (Mohammadi et al., 2020).

The resolution is maximized with (1) at the marker density that captures all linkage blocks; (2) with a population that has enough diversity to display nucleotide segregation across the genome; and (3) sufficient recombination between segregant markers to enable the detection of the marker to the causative locus.

## GWAS using structured populations

In genetics, structure is the term reserved to define the existence of stratifications in a population, where a subpopulation may differ with respect to its origin, evolutionary history, and allele frequency. Association studies in structured populations are likely to provide spurious results if the structure is not accounted for in the statistical model. Without a structure term, the model is incapable of differentiating a signal from a marker in LD to a QTL and a marker that displays higher frequency in subpopulations with higher (or lower) phenotypic means. The latter is the case when the marker tracks population structure instead of the true associations.

Key parametrizations of population structure include (1) model-based terms as derived from STRUCTURE software (Porras-Hurtado et al., 2013); (2) reduced dimensionality techniques such as principal components (Patterson et al., 2006); and (3) polygenic terms that describe the relationship among individuals (Kang et al., 2008; Xavier et al., 2016b). From those, model-based covariates and principal components are treated as fixed effects, whereas the polygenic term is random. Model-based terms are derived beforehand through clustering. Principal components are obtained from the single-value decomposition of the genotypic matrix as


M=UDS

where **M** is the genotypic matrix where rows are individuals and columns are markers, **U** is the matrix of orthogonal eigenvectors (**U**′**U** = **I**) that correspond to the principal components, **D** is a diagonal matrix with the eigenvalues, which inform how much variation of **M** is explained by each principal component, and **S** is the rotation matrix. The alternative model for genome-wide association containing either principal components or model-based covariates is commonly defined as


y=Xb+ma+e

where **y** is the vector of phenotypes, **X** is the design matrix of fixed effects containing a vector of ones and the structure term (e.g., vectors of **U**), corresponding to the principal components or model-based covariates, **b** is a vector of fixed effect coefficients including the intercept and the regression coefficients of the structure term, **m** is the vector with marker genotype information, **a** is the allele substitution effect, and **e** is the vector of residuals.

Structure modeled by the polygenic term starts with constructing the relationship matrix **G**. There are various methods to build the **G** matrix, which may entail the use of marker information, pedigree information, or a combination of both (Aguilar et al., 2011), capturing additivity, dominance and epistasis (Xu, 2013). A common choice is the linear relationship derived from genomic information, popularly known for its applications in prediction as the GBLUP model (Habier et al., 2007; VanRaden, 2008), which is computed as


$$G=\frac{MM^{\prime }}{\sum_{\text{j}=1}^{\text{P}}{\text{p}}_{j}(1-{\text{p}}_{j})}$$

where the cross product of the genotypic information matrix (**MM**′) is normalized by the sum of allele variances under Hardy-Weinberg equilibrium, defined for the j^th^ marker as Var(mj) = p_j_(1-p_j_). The genomic relationship matrix enters the genome-wide model as the covariance of the random term that describes the polygenic effect (**u**), which is assumed to be normally distributed as $u∼\text{N(}0,G\sigma_{\text{u}}^{2})$ . In prediction nomenclature, polygenic effects are referred to as genomic estimated breeding values (GEBV) and are the preferred metric for the selection of superior genotypes in breeding programs adept at genomic selection technology. The linear model for the alternative hypothesis is then defined by


y=μ+ma+Zu+e

where **Z** is the incidence matrix of individuals, and the joint variance of phenotype and random terms is defined as


$$Var\left[\begin{array}{c} y\\ u\\ e \end{array}\right]=\left[\begin{array}{ccc} V & ZGZ^{\prime }\sigma_{\text{u}}^{2} & 0\\ ZGZ^{\prime }\sigma_{\text{u}}^{2} & G\sigma_{\text{u}}^{2} & 0\\ 0 & 0 & I\sigma_{\text{e}}^{2} \end{array}\right]$$

where **V** is the variance-covariance matrix, defined as $V=ZGZ^{\prime }\sigma_{\text{u}}^{2}+I\sigma_{\text{e}}^{2}$ . For models containing random terms other than the residuals, also known as mixed effect models, the restricted likelihood (Searle et al., 2009) function is a preferred metric over the regular likelihood for the LRT because it accounts for the degrees of freedom of the fixed effects. The restricted log likelihood is defined by


$$\text{logL}(\text{X|}b,V)=\text{c}-\frac{1}{2}\text{ln}\left|V\right|-\frac{1}{2}\ln\left|X^{\prime }V^{-1}X\right|-\frac{1}{2}{\left(y-Xb\right)}^{\prime }V^{-1}(y-Xb)$$

The restricted likelihood can also be attained as a pseudorandom model (Xu et al., 2009), where all terms are considered random and fixed effect terms are assumed to have variance equal to infinity ${\text{(σ}}_{\text{b}}^{2}=\infty )$ . This leads to a simpler formulation or the restricted likelihood as


$$\text{logL}(\text{X|}b,V)=\text{c}-\frac{1}{2}\text{ln}\left|P\right|-\frac{1}{2}y^{\prime }Py$$

where the matrix **P** is a replacement of **V** ^-1^ that includes the fixed effects, as $P={\left(XX^{\prime }\sigma_{\text{b}}^{2}+V\right)}^{-1}$ , which equates to **P** = **V** ^-1^ – **V** ^-1^ **X**(**X**′ **V** ^-1^ **X**)^-1^ **X**′ **V** ^-1^. The variance components needed to estimate **V**, namely, $\sigma_{\text{u}}^{2}$ and $\sigma_{\text{e}}^{2}$ , are estimated as the values that maximize the restricted likelihood, hence referred to as the restricted maximum likelihood estimates, or “REML”. The main algorithms for solving the variance components problem are the first derivative *via* Expectation-Maximization (Harville, 1977) and the second derivative approach through average information (Johnson and Thompson, 1995). However, when the variance components are re-estimated for every alternative model, specialized algorithms such as the efficient mixed model association EMMA (Kang et al., 2008) have gained popularity. For computational efficiency, it is a common practice to use the variance components estimated for the null model in the alternative model (Aulchenko et al., 2007; Kang et al., 2010; Zhou and Stephens, 2012).

In more recent years, p values have also been driven from a linear transformation of the polygenic term (Legarra et al., 2018; Aguilar et al., 2019). This is because marker effects can be estimated from the null model as


$$\hat{\text{a}}\text{ | }\hat{\text{u}}\text{ = }{\text{MG}}^{-1}\hat{\text{u}}$$

and the p values can be directly obtained from a statistic ${\hat{\text{a}}}_{\text{j}}\;/\;{\hat{\sigma }}_{a}$ , equivalent to EMMAX, with p values obtained as


$${\left(\text{p}-\text{value}\right)}_{\text{j}}=2\left(1-\text{Φ}\left|\frac{{\hat{\text{a}}}_{\text{j}}}{{\hat{\sigma }}_{a}}\right|\right)$$

Some combination of structure parametrizations has also been proposed. Zhang et al. (2010) proposed using principal components along with a compressed polygenic term, where **G** does not express the relationship among individuals but the relationship among clusters of individuals, aiming to depict the subpopulations. However, principal components should not be combined with the non-compressed polygenic term because both parametrizations carry redundant information because the principal components can also be estimated through the eigenvalue decomposition of the genomic relationship matrix as **G = UD^2^U′**.

## Approach 2: Multiple-marker association

In this section, we present an alternative to the statistics of single marker association. These approaches are derived from methods originally proposed as prediction methods that can also serve to identify markers and genomic regions with strong associations with the trait of interest. These include the whole-genome regression methods in the Bayesian framework (Meuwissen et al., 2001) and machine learning methods (Nicholls et al., 2020).

*Whole genome regression*: Associations from whole genome regression are based on methods such as BayesC*π* (Habier et al., 2011) to infer the associations (Colombani et al., 2013) using the posterior probability of the variable selection term or the Bayes factor (Fernando and Garrick, 2013). Whole-genome regression fit models with all markers at once and have a different setup for hypothesis testing. This does not require multiple testing corrections such as Bonferroni. The BayesC*π* model is defined as


y = μ + Maδ + e

where **y** is the vector of phenotypes, **μ** is the intercept, **M** is the genotypic matrix where rows are individuals and columns are markers, **a** is the vector of allele substitution effect, **δ** is a vector of variable selection, and **e** is the vector of residuals. This model has the following probabilistic assumptions:


$$\text{μ}|\sigma_{\text{e}}^{2}∼\text{N}(\hat{\text{μ}},{\text{n}}^{-1}\sigma_{\text{e}}^{2})$$

$$a|\sigma_{\text{a}}^{2}∼\text{N(}0,\sigma_{\text{a}}^{2})$$

$$\delta |\alpha_{0},\beta_{0}∼\beta (\alpha_{0},\beta_{0})$$

$$e|\sigma_{\text{e}}^{2}∼\text{N(}0,\sigma_{\text{e}}^{2})$$

$$\sigma_{\text{a}}^{2}|\nu_{\text{a}},{\text{S}}_{a},\text{π}∼\pi^{-1}{\text{χ}}^{-2}(v_{\text{a}},{\text{S}}_{a})$$

$$\sigma_{\text{e}}^{2}|\nu_{\text{e}},\text{S}∼{\text{χ}}^{-2}(v_{\text{e}},{\text{S}}_{e})$$

The parameters are estimated as the posterior mean from the Gibbs sampler (Habier et al., 2011; Xavier et al., 2016b). The association significance can be driven by **δ** as the probability of each marker being in the model. In addition to the association, GEBVs from this model are obtained as $\hat{\text{u}}\text{ = M}\hat{\text{a}}\hat{\text{δ}}$ and the heritability as


$${\hat{h}}^{2}=\frac{{\hat{\sigma }}_{\text{a}}^{2}\sum_{\text{j}=1}^{\text{P}}\text{p}(1-\text{p})}{{\hat{\sigma }}_{\text{a}}^{2}\sum_{j=1}^{\text{P}}{\text{p}}_{j}(1-{\text{p}}_{j})+{\hat{\sigma }}_{\text{e}}^{2}}$$

*Machine learning with variable selection*: Like the approach above, this is based on fitting all markers at once in a linear model, and the main techniques utilized from association are the least absolute shrinkage and selection operator (LASSO; Tibshirani, 1996) and elastic net (Zhou and Hastie, 2005). The linear model for these models consists of


y=μ+Ma+e

with the same terms as the Bayesian whole genome regression, without **δ**. Conversely, the variable selection of LASSO and elastic net comes from the nature of the estimator of **a**. For the elastic net, the vector of effects is estimated to minimize the function


$$\text{argmin(}a)=e^{\prime }e+\psi \text{α}\sqrt{a\prime a}+\psi (1-\text{α})(a^{\prime }a)$$

where LASSO assumes *ψ* = 1 and elastic nest assumes 0< *ψ<* 1. The regression coefficients are solved *via* coordinate descent (Friedman et al., 2010). The univariate solution for the j^th^ marker is given as


{if(mj′ y˜ > 0 and mj′ y˜ − ψα < 0)→ a^j = 0if(mj′ y˜ > 0)→ a^j=mj′ y˜ − ψαmj′mj+ψ(1−α)if(mj′ y˜ < 0 and m′ y˜ − ψα > 0)→ a^j = 0if(mj′ y˜ < 0)→ a^j=m′ y˜ − ψαmj′mj+ψ(1−α)

where $\tilde{\text{y}}$ is the vector of phenotypes conditional to all except the jth markers; thus, $\tilde{y}=y-\mu -M_{-\text{j}}a_{-\text{j}}$ . The value of Λ is found through k-fold cross-validation, aiming to minimize the mean square prediction error, and GEBVs can be computed as $\hat{u}=M\hat{a}$ . Unfortunately, LASSO and elastic net do not necessarily provide a probability of associations such as a p value or Bayes factor. Associations can be inferred directly from the estimated coefficients (Waldmann et al., 2013), and an empirical significance threshold can be estimated from permutations (Doerge and Churchill, 1996).

*Machine learning with variable importance*: Semi-parametric machine learning methods do not infer any direct relationship between markers and traits. However, a general metric of association referred to as “variable importance” can be utilized as an indirect metric that provides a degree of association without revealing the nature of the association (e.g., additive, dominant, epistatic). Measurements of variable importance can be generated for support vector regressions, random forests, gradient boosting machines, and neural networks. However, there is no gold standard method to measure variable importance across machine learning methods. The use of variable importance as a genome-wide association statistic often relies on an empirical significance threshold that can be estimated from permutations (Doerge and Churchill, 1996). Approaches to generate p values *via* permutation have also been proposed for some methods, such as random forest (Altmann et al., 2010).

Among the semi-parametric machine learning methods, the random forest algorithm (Breiman, 2001) is the most popular method for genome-wide association studies (Goldstein, 2011; Brieuc et al., 2018). Random forest consists of an average prediction from a series of classification and regression trees generated with random subsets of the parameter space from bootstrapped observations. Whereas decision trees are poor predictors, the collective of multiple small trees generated at random provides robust predictions. Random forest can be described by the model (Xavier, 2021)


$$y=\frac{1}{{\text{N}}_{T}}\sum_{i=1}^{{\text{N}}_{T}}\text{T}(M_{\text{p}\in \text{P}})$$

where N_T_ is the total number of trees and T(**M**_p∈P_) is a function that represents a tree built with a random subset of markers (p ∈ P) from the genotypic matrix **M** whereas the number of trees N_T_ is at times considered a tuning parameter, higher counts provide more stable measurements of variable importance. The common metric for variable importance in random forests is the mean decrease impurity, or simply “impurity”, which corresponds to the reduction in variance for regression problems and the Gini index for classification problems.

## Approach 3: Combination of methods

Distinct GWAS approaches often provide different association results; hence, the deployment of various methods may lead to the discovery of more signals across the genome. Stronger signals are likely to be captured by multiple methods, whereas minor QTLs may be found by a specific methodology that best reflects the role of any given marker under the general architecture of the trait. Yang et al. (2018) performed genome-wide association using three types of single-marker association methods with different statistical assumptions to find the QTLs for kernel row number in corn. Going one step further, association analysis was performed deploying three distinct techniques, namely, single-marker analysis, Bayesian whole-genome regression and random forest, in the search for QTLs that control the variance components of soybeans (Xavier and Rainey, 2020). The use of multiple methods and parametrizations can be beneficial to studies seeking a multitude of signals to find consensus associations as well as alternative putative associations for follow-up investigations.

## Statistical tools for GWAS analysis

**Figure 1:**
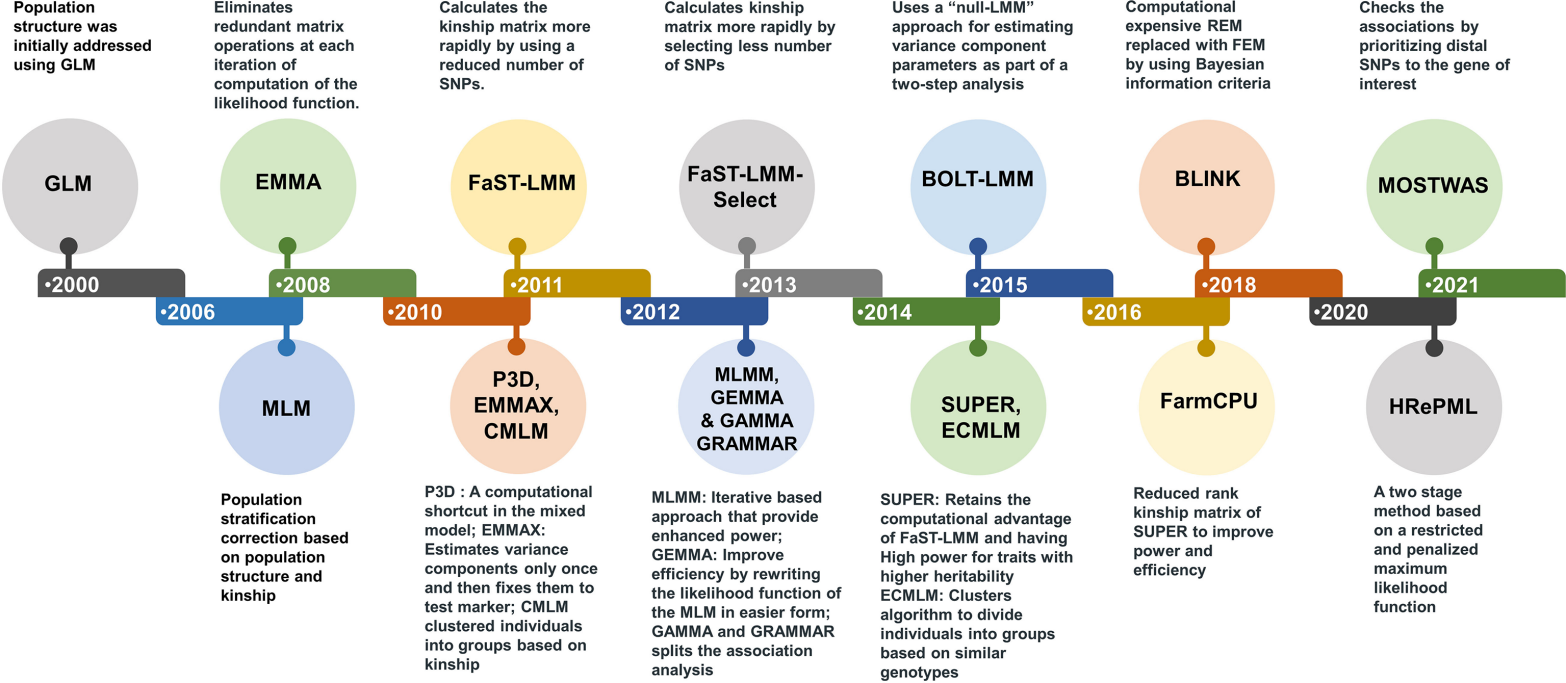
Statistical tools and model developed during last two decades. The new models developed improved the statistical power, computational speed and accuracy of detecting candidate genes or genetic loci associated with trait of interest.

Presence of population structure and genetic relatedness lead to detection of false positives in association studies. To overcome these limitations, general linear model (GLM) and mixed linear model (MLM) were used. MLM has been the most flexible and strong statistical tool for managing population structure and family relatedness (kinship; Yu et al., 2006). During recent past, several statistical tools/models evolved for addressing constraints for improving the accuracy, speed and power of detecting associations (Li et al., 2014). To improve the efficiency of solving MLM equations, many approaches have been introduced. For instance, efficient mixed-model association (EMMA) was the first of these to be designed, which enhanced computing speed by eliminating redundant matrix operations (Kang et al., 2008). Other methods like EMMA expedited (Kang et al., 2010) and population parameters previously determined (P3D) (Zhang et al., 2010), enhanced computational speed using approximation or using computational shortcuts in mixed model. Factored spectrally transformed linear mixed models (FaST-LMM) as well as genome-wide efficient mixed model analysis (GEMMA) (Lippert et al., 2011; Zhou and Stephens, 2012), both improved methods increase efficiency by rewriting the MLM’s likelihood function in a more evaluable format (**Figure 1**). Using clustering algorithms, an improved method termed the compressed MLM (CMLM) has been developed to cluster individuals into groups which improve the statistical power (Zhang et al., 2010). Further improvement in CMLM with higher statistical power was achieved through this enriched compressed MLM (ECMLM) method (Li et al., 2014). Multi-locus GWAS approaches outperform single-locus GWAS methods by using many markers in the model as variables at the same time. The multi-locus mixed model (MLMM) was the first multi-locus GWAS approaches. Bayesian information and LD iteratively nested keyway (BLINK) (Huang et al., 2018) and fixed and random model circulating probability unification (FarmCPU) (Liu et al., 2016), both are the multi-locus approaches that are based on MLMM methods. FarmCPU is consider the best multi locus GWAS approach and it controls both false positives and false negatives (Kaler et al., 2020). There are some challenges in the GWAS for polyploidy species (Garreta et al., 2021). To overcome these challenges only few software packages like GWASpoly and SHEsis (Rosyara et al., 2016; Shen et al., 2016) that accept only polyploidy genomic data were developed. In addition, to tackle these challenges, a multi GWAS tool is being developed that runs GWAS analysis for both diploid and tetraploid species simultaneously utilizing four software packages (Garreta et al., 2021). Development of improved model to reduce the challenges like population structure and relatedness is continuing to be an important research topic.

## Advances in GWAS analysis

**Figure 2:**
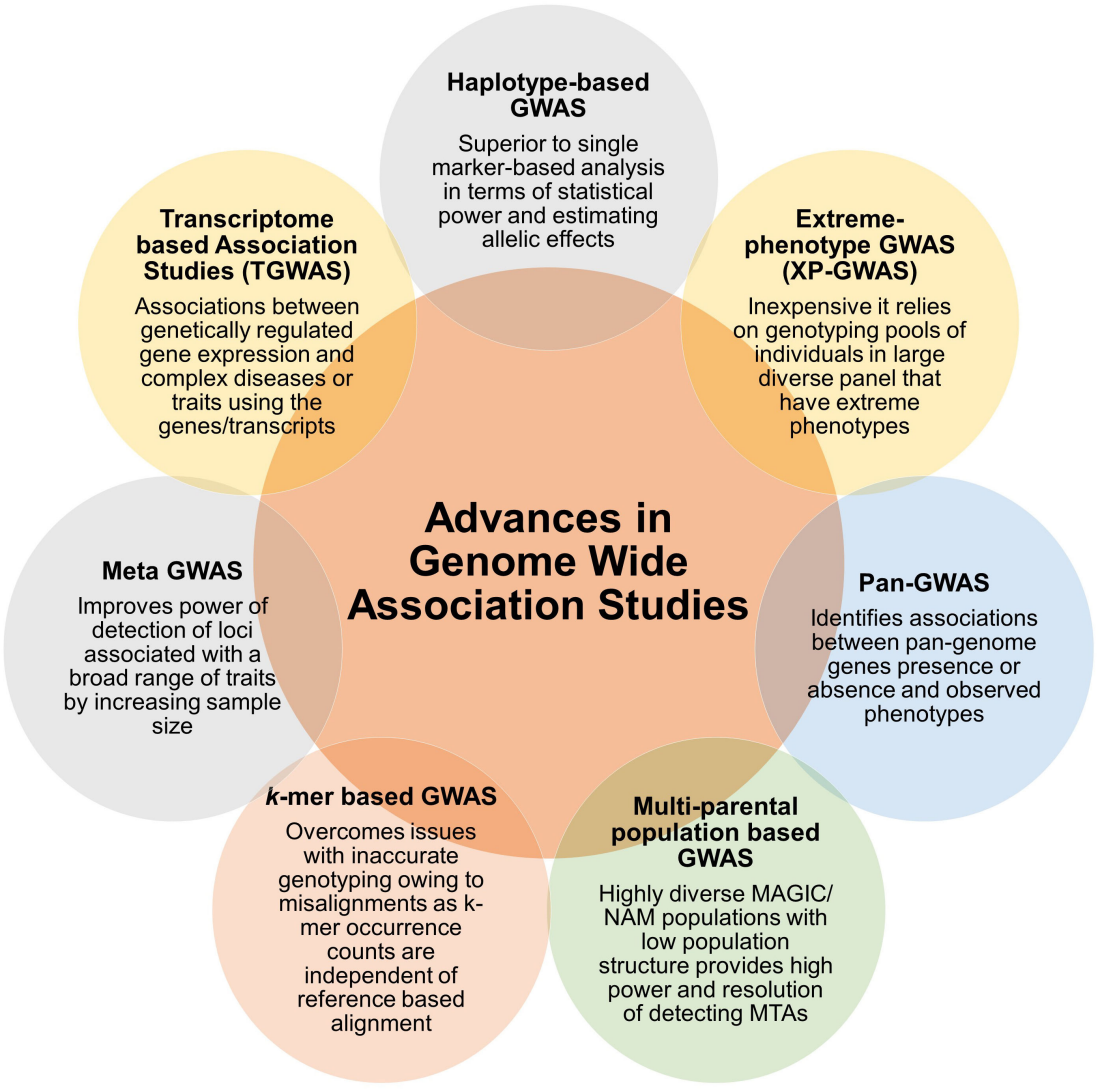
Summary of advances in association analysis. Different types of GWAS approaches are arranged in the chronological order, starting with GWAS based on halpotypes, extreme phenotypes, pangenomes, multi-parent populations, *k*-mers, meta data and transcriptomes. Key feature or major advantage of the approach is also mentioned.

During recent past, different variants in associations studies have emerged that use halpotypes, extreme phenotypes, pangenomes, multiparent populations, *k*-mers, meta data and transcriptomes that improved the efficiency and power of identification of significant MTAs in crop species or animal systems (**Figure 2**). However only a few of these association approaches were used for identification of genetic loci and candidate genes associated with diseases. We presented an account of these approaches, deploying one or more of these approaches will further enhance the fine mapping of complex diseases in plants and help in resistance breeding.

## Haplotype based GWAS

With availability of draft genomes and high to low coverage sequencing of several germplasm lines in different crops species, identification and use of superior haplotypes has been gaining importance in breeding climate smart crop varieties (Sinha et al., 2021; Varshney et al., 2021). Haplotypes are non-random association of alleles that inherit together and dissociation of haplotypes is low and mutation rate is very low in case of haplotypes. Hence haplotypes will be superior over SNPs for association studies (Qian et al., 2017). Association studies based on GWAS approach can overcome limitations associated with SNPs and boost the resolution of genomic mapping. Haplotype based GWAS has been successfully used in mapping agronomically important traits and abiotic stresses (Sehgal et al., 2020; Helal et al., 2021; Zhao et al., 2022b). Nevertheless, haplotype based GWAS was also deployed to identify candidate genes in some crop species. For instance, in case of wheat, a comparative GWAS analysis was conducted for leaf rust resistance based on SNPs and haplotypes and reported a greater number of associations using haplotypes (69 MTAs) compared to SNPs (25 MTAs) based GWAS. Further analysis using haplotypes identified more genomic regions and additional functional genes (*Lr10* and *Lr1*) compare to SNPs based analysis (Liu et al., 2020). Therefore, haplotype based GWAS has potential that can be exploited for identification of genetic loci associated with key diseases in crop plants.

## Extreme phenotype GWAS (XP-GWAS)

Association mapping uses historical recombinations to identify genetic loci or candidate genes associated with a complex trait, and provides maximum resolution than would be possible with similar sized mapping populations using association analysis (Alqudah et al., 2020). However, a major disadvantage in the case of QTL mapping and association studies is the need for extensive genotyping and phenotyping data, which can be costly for large populations. As a novel solution to this challenge, is extreme phenotypes based GWAS that does not require genotyping a large number of individuals (XP‐GWAS; Yang et al., 2015). Extreme phenotypes are a group of extremely resistant and susceptible lines for disease response that have been selected using a simple approach for determining disease symptoms. It mainly depends on the pool size, selection intensity, precision of phenotyping, genome-wide marker distribution, and read depth of the sequence, and these factors may affect the power of XP‐GWAS. In addition, it relies on variations in allele frequencies of markers in linkage disequilibrium with the QTL of interest in the pool. For each trait of interest, a new XP-GWAS experiment must be conducted, and it may identify fewer marker-trait associations than the conventional GWAS method because pooling introduces stochastic and uncertainties. XP-GWAS is very much beneficial in species for which there are no significant genotyping resources available, such as wild crops, orphan crops, and uncharacterized species (Yang et al., 2015). Extreme phenotypes can be used to determine QTLs and screen candidate genes quickly. Cui et al. (2021) conducted an experiment on XP‐GWAS and 145 trait-associated variants for kernel row number traits were identified in maize at a false discovery rate of 0.05. These identified associations are somewhat less than the number obtained (260) by the conventional GWAS approach, but this lacunae is counter balanced by a considerable reduction in the cost of genotyping (Yang et al., 2015). Extreme phenotypes bulk were used and identified genomic loci *rp1* associated with resistance to goss’s wilt of maize (Hu et al., 2018). Novel *pi21* haplotypes were identified, confirming resistance to rice blast disease, by using a combined approach of bulked segregant analysis and genome sequencing mapping (Liang et al., 2020). Combining the approach of extreme phenotypes with GWAS provides higher resolution with cost-effective candidate gene identification; additionally, it improves genomic information for particular traits.

## Pan-GWAS

In addition, Pan-GWAS is an important and useful approach to identify the number and nature of the mutations encountered in the different species of the organisms. Using this approach, we can identify the ancestors or the source of the particular gene responsible for different resistance/tolerance action. Diverse collections of genes from the different sources/species conferring increased potential for accuracy allelic variants of these genes distinguish carriage from invasive strains. Gene locations identified from Pan-GWAS can tell us the information about even/random spreading of DNA sequences among the chromosomes (Gori et al., 2020; Gupta, 2021). Pan-GWAS is mostly used in the microbial study, as the genome of different strains of microorganism can be sequenced easily. Same approach can be used in the crop species, by using previously sequenced data present in the databases. Pan-GWAS approach can also be used in the disease resistance by identifying the nature and origin of the different disease strains of the microorganisms. Pan-GWAS analyzed 42 genes of *Pantoea ananatis*, among those 28 newly discovered genes that were not previously associated with pathogenicity in onion (*Allium cepa*) (Agarwal et al., 2021). More than 10 million SNPs, 99000 small indels and 16000 presence/absence variations as well as 17000 copy number variations were identified, containing leucine rich repeats, PPR repeats and disease resistance *R* genes possessing diverse biological functions in sorghum by re-sequencing two sweet and one grain inbred lines (Zheng et al., 2011). Scoary and Roary are the tools which are widely used for Pan-GWAS analysis. Scoary is a web-tool for scoring the associations between phenotypes and the components of pan-genome. The algorithm of the Scoary uses population stratification with the minimum potential assumptions of evolutionary processes and sorted genes by strength of trait association (Brynildsrud et al., 2016). Roary is a tool which is used to develop the large-scale pan genomes by identifying the core and accessory genes within the representative genome. It makes construction of the pan genome of thousands of prokaryote samples possible with the great accuracy (Page et al., 2015).

## GWAS based on multiparent populations

Multiparent populations will have high power and resolution for fine mapping of disease resistance. MAGIC and NAM populations possess high genetic diversity, minimal population structure, large number of QTLs, and serve as sources of information for breeding and pre-breeding programs (Scott et al., 2020). Using MAGIC population developed from eight founder lines, genotypic and phenotypic interactions were found to be significant for Septoria tritici blotch (STB) and PM disease scores in wheat. The GWAS-assisted genomic prediction (GP) ranged within 0.53-0.75 for STB and 0.36-0.83 for PM. In case of rice, using disease resistance data of 144 MAGIC Plus lines and a total of 14,242 SNPs, 57 significant genomic regions with a −log10 (P value) ≥ 3.0 were reported. Of which, two major loci (qBLB11.1 and qBLB5.1), were identified for bacterial leaf blight (BLB) resistance and *Pi5(t)*, *Pi28(t)*, and *Pi30(t)* genes were identified for blast resistance (Descalsota et al., 2018). Downy mildew, caused by the oomycete *Peronospora effuse* has been fine mapped in case of spinach (*Spinacia oleracea*) and the most promising candidate genes *Spo12784* and *Spo12903* near the *RPF1* locus were reported (Bhattarai et al., 2021).

## Pan-MAGIC GWAS

The high-quality genomes enabled the identification of numerous complex variations that cannot be detected by simply mapping the short reads to a single genome and the graph-based genome offers a new platform to map short read data to determine the genetic variations at the pan-genome level (Rakocevic et al., 2019). Two MAGIC populations (i) a subset of 124 lines of the MAGIC population previously obtained by crossing eight tomato plants selected to include a wide range of genetic diversity and (ii) the GWAS diversity panel consisting of 136 accessions of small fruit tomato were used in the GWAS study to identify 25 QTLs interspersed across the genome responsible for tocopherol biosynthetic pathway that modulates salicylic acid accumulation against the basal resistance to *Pseudomonas syringae* in Arabidopsis. (Burgos et al., 2021). Similarly four multi-parent populations: I MAGIC (8 *indica* parents); MAGIC plus (8 *indica* parents with two additional rounds of 8-way F_1_ inter-crossing); japonica MAGIC (8 *japonica* parents); and Global MAGIC (16 parents - 8 *indica* and 8 *japonica*) were created to directly and indirectly employ the highly recombined lines in breeding programs, for studying the interactions of genome introgressions and chromosomal recombination and to fine map the QTLs for several characteristics (Bandillo et al., 2013) (**Table 2**). In Pan-MAGIC approach a reference genome developed by combining the accessory and core genome of founder parents can be used for variant calling and subsequent genome wide association studies. A multi-parent population combines several founders, therefore use of a single reference genome results in reference bias and there are possibilities of loosing the variants from accessory genome. Therefore, a Pan-genome developed from the founder parents of respective multi-parent population (NAM, MAGIC) can be used as a reference and variant calling, this approach can capture the alleles from each founder parent segregating in multi-parent populations.

**Table 2:** Summary of multi-parent populations used for identifying MTAs for disease resistance in different crops.

	Founder parents	Traits	Reference
Maize	One eight-way cross was made and was randomly mated up to six generations to develop 672 highly homozygous RILs	Corn borers	Jimenez-Galindo et al. (2019)
Rice	Eight *indica* parents selected from the Asian *indica* pool and are intercrossed to produce 1328 S_7_ lines	Blast and bacterial blight	Bandillo et al. (2013)
	Eight founder lines were intercrossed to derive eight-way crosses and then selfed to obtain F8 generations to get 2100 AILs (advanced intercross lines). At S_4_ 200 AILs and S_6_-S_8_ 340 AILs randomly chosen for screening	Bacterial leaf streak and bacterial blight	Bossa-Castro et al. (2018)
	144 MAGIC Plus lines	Blast and bacterial leaf blight	Descalsota et al. (2018)
	391 MAGIC *indica* rice accessions	Blast and stripe rust	Satturu et al. (2020)
Wheat	Eight winter wheat cultivars were intercrossed to generate 394 F_6:8_ RILs	Powdery mildew	Stadlmeier et al. (2018)
	Eight elite winter wheat founder lines were intercrossed up to three generations and then selfed multiple times to generate 1000 RILs	Leaf blotch and glum blotch	Lin et al. (2020)
Cotton	12 founder lines were intercrossed up to F_7_ through SSD and produced 1500	Disease and pest resistance genes	Li et al. (2016b)
Tomato	Eight tomato founder lines were crossed to develop 400 G10 stable lines through SSD methods	Several resistance genes	Campanelli et al. (2019)
Cowpea	Eight selected founder parents were intercrossed to (eight-way crosses derived from 6 pedigree funnels) produce 305 MAGIC F_8_ RILs	Biotic and abiotic stresses	Huynh et al. (2018)

## Pan-NAM GWAS

NAM has huge possibility for studying quantitative traits and associated genomic regions used to speedy discovery of candidate genes and markers within the genome (Gangurde et al., 2019). Multiple NAM populations can be used for dissecting genetic control of different complex quantitative traits and associated genomic regions in different genome and individuals. Pan-NAM GWAS can be used to identify genetic contribution of the sub-genomes in the development of particular trait. Using HEB-25 NAM population, Pan-NAM GWAS allowed to interrogate 25 different wild barley genomes, giving a rich allelic diversity and the BC_1_S_3_ genetic structure. The choice of multiple NAM lines justified the strong QTL effects and the identification of multiple QTL hotspots (Sharma et al., 2018). To reveal the usefulness and power of this tool, two NAM populations, were used and two high-density SNP-based genetic maps were constructed with 3341 loci and 2668 loci. The QTL analysis identified 12 and 8 major effect QTLs but in case of GWAS analysis was identified 19 and 28 highly significant SNP trait associations (STAs) in NAM_Tifrunner. Eleven and seventeen STAs were identified in NAM_Florida-07 for pod weight and seed weight, respectively (Gangurde et al., 2020). Considerable overlaps between the QTL identified and grain size GWAS signals in rice and maize, and the orthologues genes for grain size from rice and maize, showed the common genetic architecture underlying these characters among these cereal crops (Tao et al., 2019).

## GWAS using sequencing reads/*k*-mer

Association analysis has some limitation such as knowledge about reference genome for SNP calling (identified association in a region which is not in the reference genome is difficult), structural variants (Indel, copy number variations etc.) are ignored in GWAS studies and the rare variant associated with phenotype might be ignored. To overcome these limitations GWAS can be use sequences of nucleotide residues called it as *k*-mer, as a genotyping data (Rahma et al., 2018) to find the causal variant. It is an alignment free method for association studies. In maize *k*-mers were used in GWAS analysis for cob and kernel color traits and also identify associated *k*-mers efficiently (He et al., 2021). In another study *k*-mers based reference free GWAS analysis was conducted in soybean and identified four genomic loci for seed pigmentation (Kim et al., 2020). Collectively, it is suggested that, *k*-mers based GWAS may be an alternative approach for identifying genomic regions or genes for economically important traits like disease resistant.

## Meta GWAS

Meta GWAS analysis is a method of utilizing the results of previous studies to improve the power and resolution of association increasing sample size and by examining more variants (Zeggini and Ioannidis, 2009). Statistical approaches like METAL can be used for analyzing the results from independent studies (Willer et al., 2010). Meta GWAS analysis has been used for dissecting complex traits in human (Xue et al., 2022) as well as in crop species (Zhao et al., 2019; Fikere et al., 2020; Shook et al., 2021). In term of canola, Meta-analysis was performed for identifying resistance genes to blackleg disease and identified 79 genomic regions associated with 674 SNPs that conferring potential resistance to disease, among these 53 regions were novel (Fikere et al., 2020). In case of soybean, Meta-GWAS analysis based on 76 independent studies enhanced statistical power for robust detection of loci associated with a broad range of trait.

## Transcriptome based GWAS

Transcriptome based GWAS association approach investigates associations between genetically regulated gene expression and complex diseases or traits using the genes/transcripts. TWAS has gained popularity during last five years due to its ability to reduce multiple testing burden and has been extensively used in fine mapping different traits in humans. With the advent of single-cell sequencing, chromosome conformation capture, gene editing technologies, and multiplexing reporter assays, we are expecting a more comprehensive understanding of genomic regulation and genetically regulated genes underlying complex diseases and traits in the future. Recently, in cotton a combinatorial approach of GWAS, QTL-seq and transcriptome-wide association studies was used to discover candidate genes and developed KASP marker for verticillium wilt resistance in cotton (Zhao et al., 2021). 69 candidate genes related to plant hormones such as MAP kinase, a PR5-like receptor kinase, and heat shock proteins associated with Fusarium ear rot caused by *Fusarium verticillioides* were identified using GWAS and validated by comparing the transcriptomes (Yao et al., 2020). Transcriptome wide association analysis for southern rust of maize identified eQTLs on Chr2:231,271,050 one gene Zm00001d007424, and on Chr4:78,851,667 was identified as a cis-eQTL of three genes: *Zm00001d050283*, *Zm00001d050284*, and *Zm00001d050293* (Sun et al., 2022). A transcription factor REPLUMLESS was identified contributes to both disease resistance against hemi-biotrophic bacterial pathogen *Pseudomonas syringae* and plant growth in Arabidopsis (Xu et al., 2022).

## Future outlook

**Figure 3:**
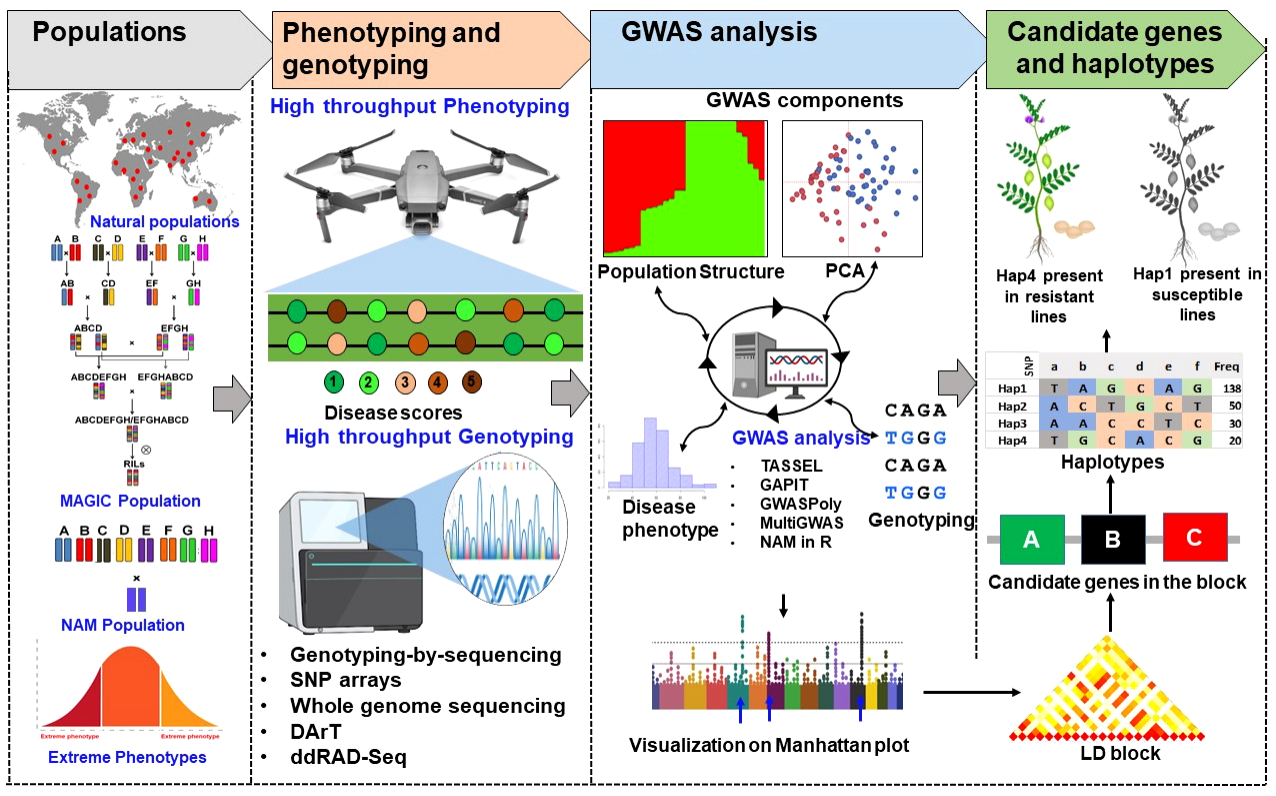
Illustration of genome-wide associations studies to identify genes associated with disease resistance. The partially structured (NAM and MAGIC) and unstructured populations (germplasm lines, association panels) can be used for high throughput phenotyping and genotyping to perform high resolution association mapping with advance tools for genome wide association analysis (GWAS). The peaks identified in GWAS analysis can be used for identification of LD blocks. Each LD block includes one or few candidate genes associated with the trait can be used for validation or development of diagnostic markers for genomics associated breeding. The validated genes can be further used for identification of haplotypes for disease resistance or disease susceptibility.

Identification of genetic loci or candidate genes is key to trait improvement in breeding programs (**Figure 3**). Rare variant, synthetic associations, small effects size, improving the choice of GWAS model, genetic heterogeneity and unexpected LD remain challenges to increase knowledge of complex traits (Cortes et al., 2021). Synthetic associations are one of the major problems that mislead GWAS results, non-associated SNPs also shows significant associations with trait of interest, allelic heterogeneity may be the major cause for this problem. Even if there is no allelic heterogeneity, rare alleles can also cause synthetic associations. In addition, amount of input data is one of the important factors that influence the statistical reliability of GWAS (Yan et al., 2018). Therefore, selection of appropriate GWAS programs according to input data is challenging and need to be standardized for improving reliability. Continuous efforts are being made by scientific community to improve the efficiency of the statistical models in detecting the loci or genes associated with key traits. Many new statistical models have been created to evaluate rare variants, by combining neighboring rare variants and examining their combined effect (Lee et al., 2014).

Meta-GWAS has emerged as a major strategy of dissecting traits to improve the strength of single-marker GWAS and enables to find the most effective stable loci spanning space and time while eliminating false positives (Evangelou and Ioannidis, 2013). In addition, constructing haplotypes between nearby SNPs on a chromosome is another way to improve the power of GWAS (Sehgal et al., 2020). High accuracy of GWAS largely depends on selection of an appropriate statistical model to reduce false positive results. In general, there is no universal model which gives best GWAS result to dissect complex traits, but each model has its own advantages compared to other models and best suitable model for GWAS. It is good to use MLM approaches to scan individual SNPs in the genome as well as other multi-locus methods to scan the genome. In terms of additional identified genomic regions using multi-locus methods, these regions must examine if the genome-wide marker coverage was appropriate so that adequate estimation of polygenic effect of population structure and kinship. GWAS can explain about 30-40% phenotypic variation of a trait, the cause of rest 60% phenotypic variation can be achieve by metabolome wide association analysis (MWAS), protein wide association analysis (PWAS) and transcriptome wide association analysis (TWAS) (Weckwerth et al., 2020). Genome wide association studies based on the multi-parent populations should use a pan-genome as a reference developed from the core and accessory genomes of founder parents to avoid the reference bias. In PAN-NAM or PAN-MAGIC genome-wide association studies the diversity from all the parents can be captured, while, in GWAS based on single reference genome we can’t capture maximum allelic diversity.

## Author contributions

MT and CZ conceived the idea; SG, AX, YD, UJ, SK, RK, MR, SC, DE, RM, RZ, LC, HS, MP, SP, VM, UR, BG, NG, VS, XW, CZ, and MT – contributed to writing the review; All authors read and approved the review article.
